# Supplementary material for: Inhibiting endocytosis in CGRP+ nociceptors attenuates inflammatory pain-like behavior
Source: Nat Commun. 2021 Oct 4;12:5812. doi: 10.1038/s41467-021-26100-6 (PMC8490418; doi:10.1038/s41467-021-26100-6)
Supplement: Supplementary file 1 — Supplementary Information [file 41467_2021_26100_MOESM1_ESM.pdf]

Supplementary Information

**Supplementary Table 1 –di-leucine based myristoylated N-terminal peptides**	Protein derivation	Sequence
Ap2 inhibitor peptide	Human CD4	RM**S***EIKRLL*SE
P1	Human EGFR	RLR***T****LRRLL*QE
P2	Human KCNT1	RLEPN*DIVYLI*RS
P3	Human CD3	RAS*DKQTLL*PNQ
P4	Human CD3	RA**S***DKQTLL*PNQ
Scrambled sequence	Human CD4	IERLSEM**S**LRK

Underline-D/EXXXLL(I) AP2 binding motif
Bold –phosphorylated residue

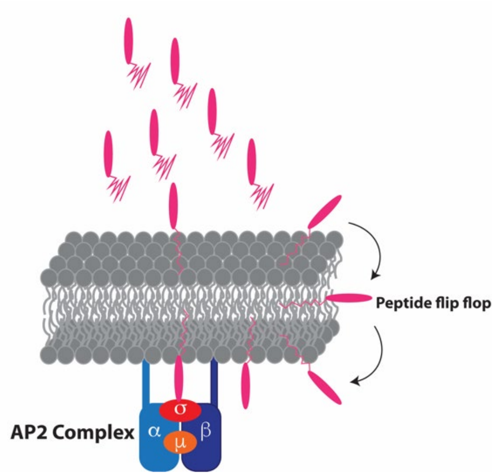

Supplemental Figure 1. Lipidated small peptides insert into the membrane and flip flop to the inner leaflet of the cell. The AP2 complex is a multimeric protein that recognizes and internalizes cargo by clathrin-mediated endocytosis. Myristoylation of the amino terminal of small peptides allow peptides to partition on the outside of the membrane and flip flop to the inner side and interact with potential intracellular, membrane associated targets.

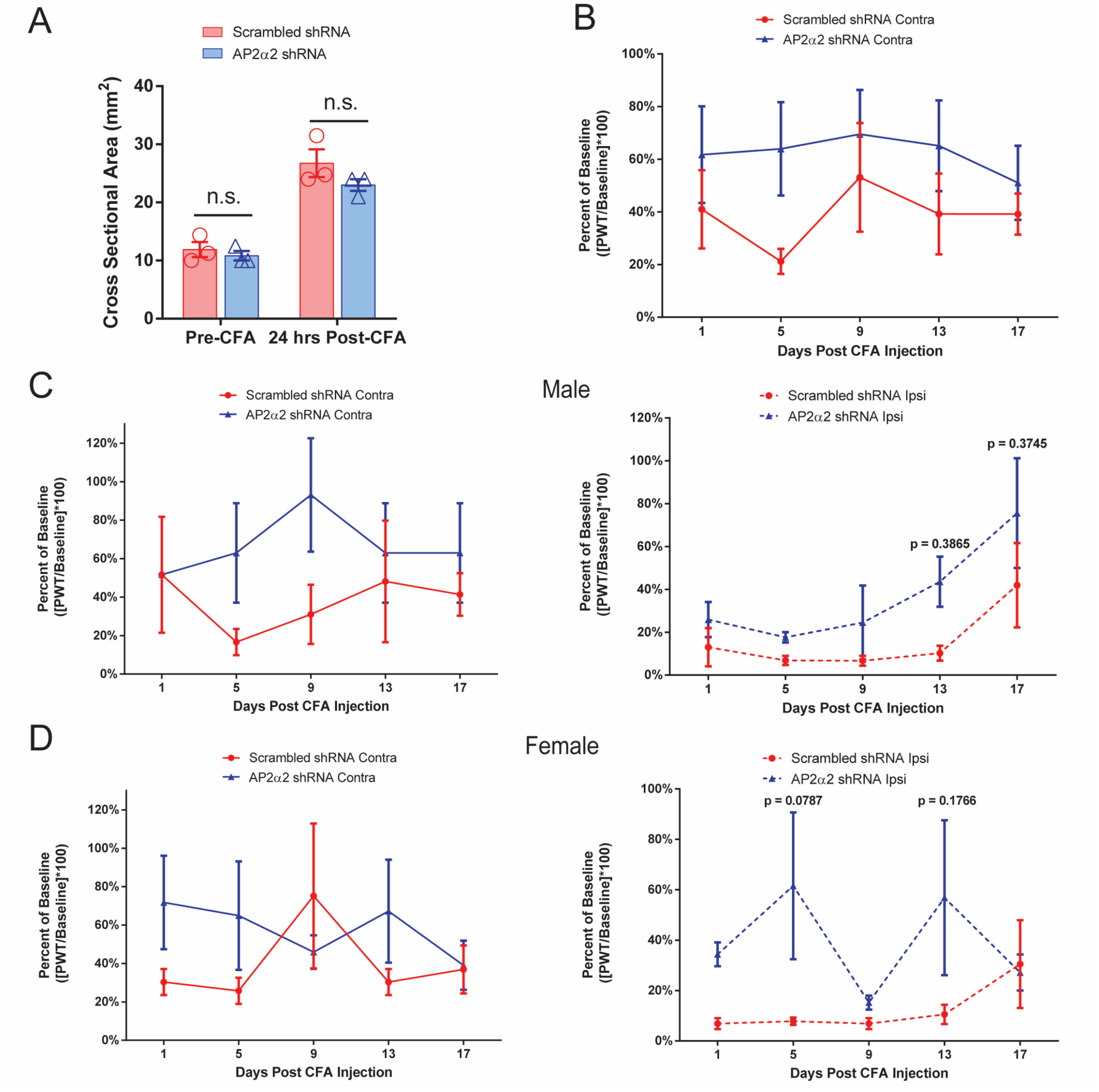

Supplemental Figure 2. shRNA mediated knockdown of AP2α2 does not significantly impair CFA-induced ipsilateral swelling or contralateral von Frey paw withdraw threshold. (A) Cross-sectional area of ipsilateral hind paws 24 hours before and following CFA injection. Measurements were taken from awake C57BL/6 mice with calipers. Width and height were taken from the widest part of the hind paw. Each group (scrambled shRNA and AP2α2 shRNA) has n = 3. The same animal was measured before and following CFA injection. Data is represented as cross sectional area mean ± S.E.M. and analyzed using 2-way ANOVA statistical test with Bonferroni correction. (B – D) von Frey filament testing of animals in a CFA-induced model of chronic inflammatory pain. Data is represented as mean PWT (as a percent of baseline PWT) ± S.E.M. Statistical significance was determined using repeated measures 2-way ANOVA statistical test with Bonferroni correction (B) Contralateral PWT. Data is complimentary to data presented in figure 2B. (C) [Left] Contralateral PWT for male animals injected with either scrambled shRNA (n = 4) or AP2α2 shRNA (n = 4). [Right] Ipsilateral paw. (D) [Left] Contralateral PWT for female animals injected with either scrambled shRNA (n = 4) or AP2α2 shRNA (n = 4). [Right] Ipsilateral paw.

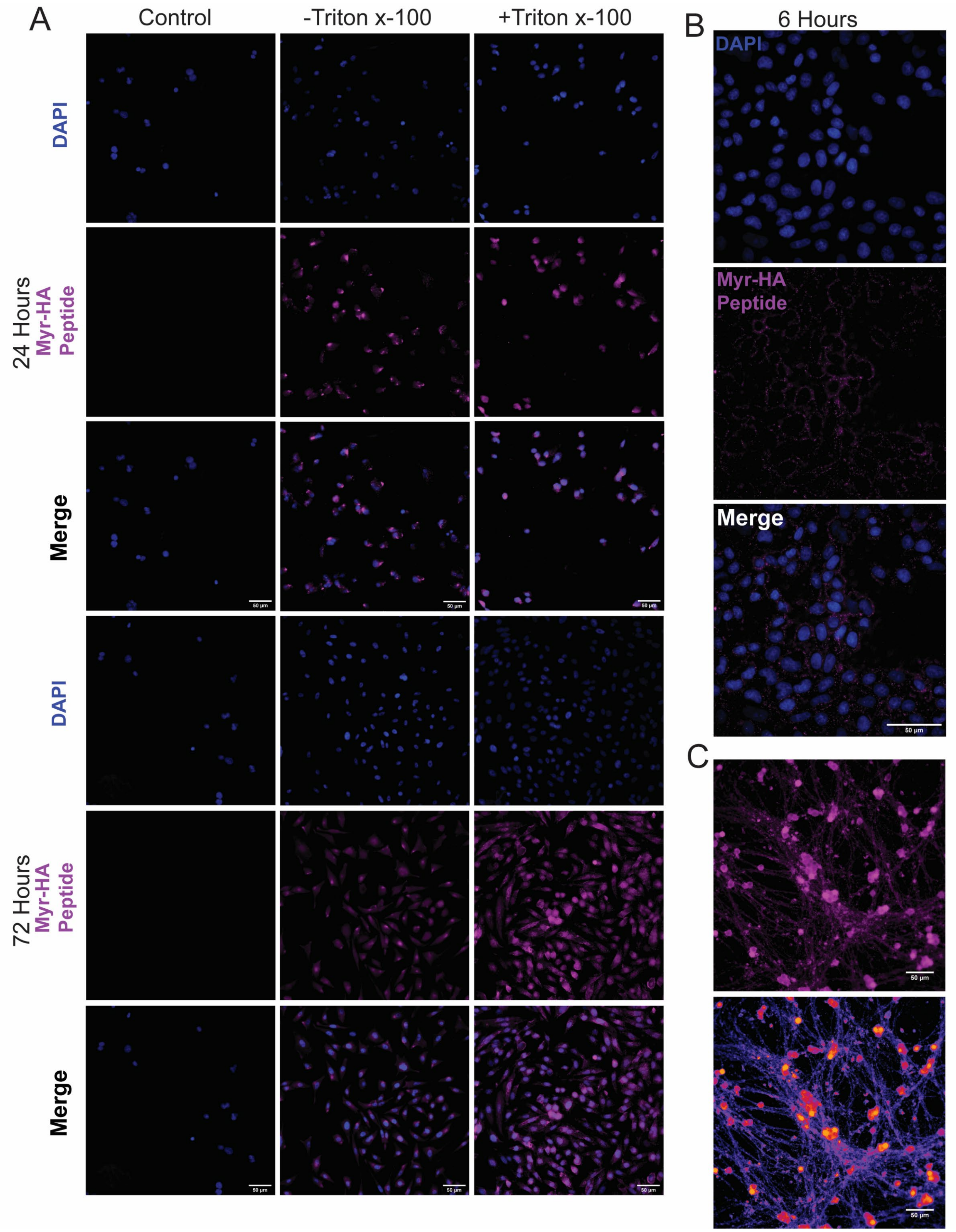

Supplemental Figure 3. Lipidated HA-peptide exhibits robust stability in mitotic cells and post-mitotic neurons. CHO cells were cultured in appropriate conditions for at least 2 days following seeding. On the day of experimentation, the media was changed, and replaced with growth media supplemented with the HA-peptide (10 μM). The cells were incubated in the HA-peptide supplemented media for 3 hours, at which point the media was removed, the cells were washed with PBS

and allowed to grow until collection. Cells were fixed and stained with a HA-specific antibody (A) Representative images from cultured CHO cells exposed to the HA-peptide under varying conditions of permeabilization and time points. '-Triton x-100' is indicative of extracellular-only HA-peptide, whereas '+Triton x-100' is indicative of total HA-peptide immunoreactivity. Using permeabilizing and non-permeabilizing conditions demonstrated that the HA-peptide can flip-flop continuously from either side of the cell membrane and is therefore primarily membrane-delimited. (B) High magnification oil-immersion image illustrating the localization of the HA-peptide in the membrane of CHO cells 6 hours following incubation. (C) Representative image of HA immunoreactivity in cultured embryonic DRG neurons 3 days following initial exposure to the HA-peptide. [Bottom] A look-up-table transformation of the top image depicting intensity of staining.

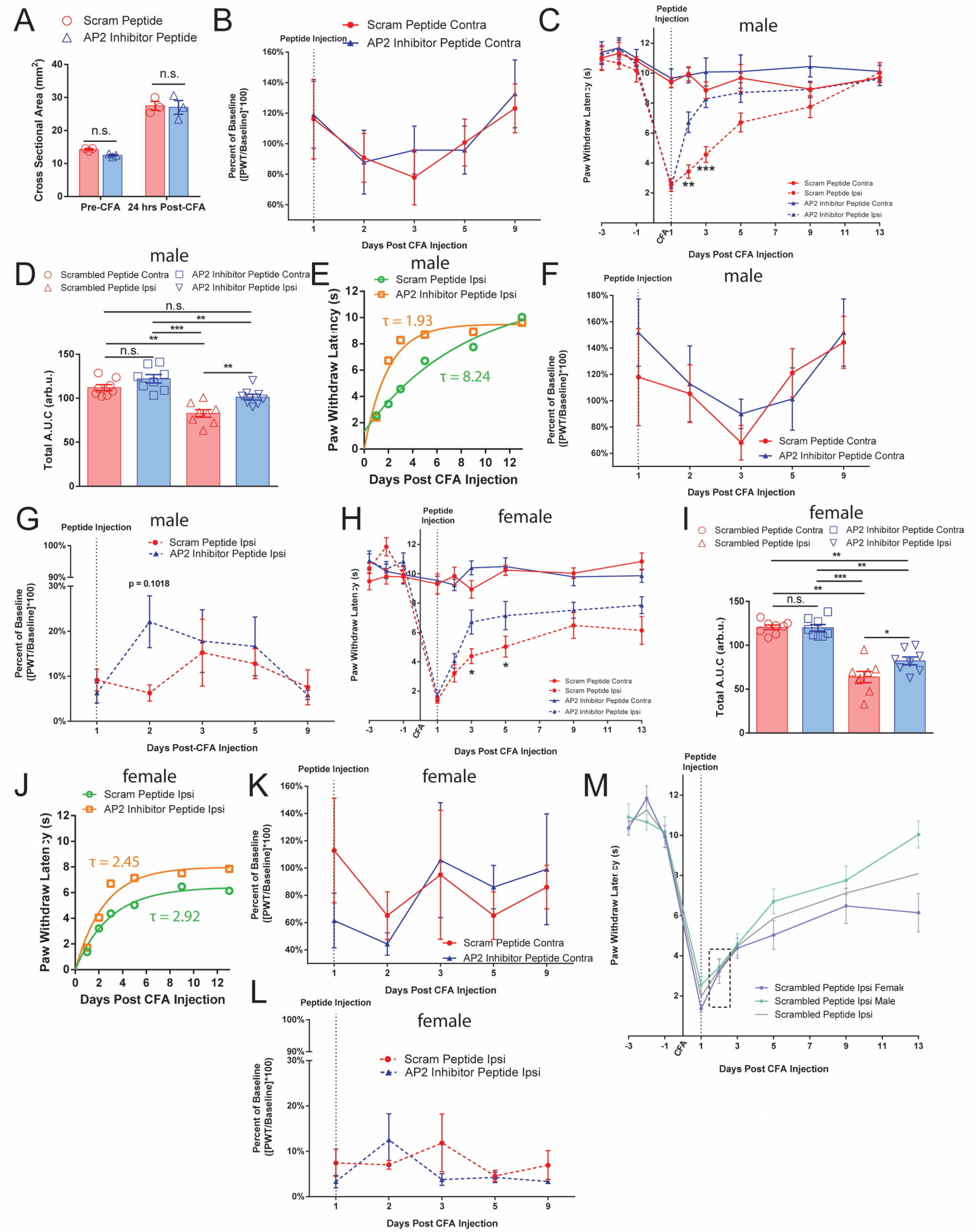
A
Scram Peptide
AP2 Inhibitor Peptide
Cross Sectional Area (mm2)
n.s.
n.s.
Pre-CFA
24 hrs Post-CFA
B
Scram Peptide Contra
AP2 Inhibitor Peptide Contra
Peptide Injection
Percent of Baseline ([PWT/Baseline]*100)
Days Post CFA Injection
C
male
Peptide Injection
Paw Withdraw Latency (s)
Scram Peptide Contra
Scram Peptide Ipsi
AP2 Inhibitor Peptide Contra
AP2 Inhibitor Peptide Ipsi
Days Post CFA Injection
D
male
Scrambled Peptide Contra
Scrambled Peptide Ipsi
AP2 Inhibitor Peptide Contra
AP2 Inhibitor Peptide Ipsi
Total A.U.C (arb.u.)
E
male
Scram Peptide Ipsi
AP2 Inhibitor Peptide Ipsi
τ = 1.93
τ = 8.24
Paw Withdraw Latency (s)
Days Post CFA Injection
F
male
Peptide Injection
Scram Peptide Contra
AP2 Inhibitor Peptide Contra
Percent of Baseline ([PWT/Baseline]*100)
Days Post CFA Injection
G
male
Peptide Injection
Scram Peptide Ipsi
AP2 Inhibitor Peptide Ipsi
p = 0.1018
Percent of Baseline ([PWT/Baseline]*100)
Days Post-CFA Injection
H
female
Peptide Injection
Scram Peptide Contra
Scram Peptide Ipsi
AP2 Inhibitor Peptide Contra
AP2 Inhibitor Peptide Ipsi
Paw Withdraw Latency (s)
Days Post CFA Injection
I
female
Scrambled Peptide Contra
Scrambled Peptide Ipsi
AP2 Inhibitor Peptide Contra
AP2 Inhibitor Peptide Ipsi
Total A.U.C (arb.u.)
J
female
Scram Peptide Ipsi
AP2 Inhibitor Peptide Ipsi
τ = 2.45
τ = 2.92
Paw Withdraw Latency (s)
Days Post CFA Injection
K
female
Peptide Injection
Scram Peptide Contra
AP2 Inhibitor Peptide Contra
Percent of Baseline ([PWT/Baseline]*100)
Days Post CFA Injection
L
female
Peptide Injection
Scram Peptide Ipsi
AP2 Inhibitor Peptide Ipsi
Percent of Baseline ([PWT/Baseline]*100)
Days Post CFA Injection
M
Peptide Injection
Scrambled Peptide Ipsi Female
Scrambled Peptide Ipsi Male
Scrambled Peptide Ipsi
Paw Withdraw Latency (s)
Days Post CFA Injection

Supplemental Figure 4. Pharmacological inhibition of endocytosis precipitated sexually dimorphic pain-like responses to thermal stimuli in a model of chronic inflammatory pain. (A) Cross-sectional area of ipsilateral hind paws 24 hours before and 24 hours following CFA injection. Measurements were taken from awake C57BL/6 mice with calipers. Width and height were taken from the widest part of the hind paw. Each group (scrambled and AP2 inhibitor peptidomimetics) has n = 3. The same animal was measured before and following CFA injection. Data is represented as cross sectional area mean ± S.E.M. and analyzed using 2-way ANOVA statistical test with Bonferroni correction $p < 0.05$. (B) von Frey filament testing of animals in a CFA-induced model of chronic inflammatory pain. Data is represented as mean PWT (as a percentage of baseline PWT) ± S.E.M. Statistical significance was determined using repeated measures 2-way ANOVA statistical test with Bonferroni correction $p < 0.05$. Data is complimentary to data presented in figure 4G. (C – G) Complied behavioral data for male animals during CFA model of chronic inflammatory pain. (C) Thermal sensitivity of male mice injected with scrambled (n = 8) and AP2 inhibitor (n = 8) peptide after CFA. Males exhibited rapid return to baseline after AP2 inhibitor peptide injection. Data is presented as mean PWL ± S.E.M. Significance determined using repeated measures 2-way ANOVA with Bonferroni correction $p < 0.01$; **, $p < 0.005$; ***. Ipsilateral Scrambled peptide vs Ipsilateral AP2 peptide: Day 2 p-value = 0.0011, Day 3 p-value = 0.0002. (D) Recovery A.U.C. for male mice under experimental conditions; scrambled peptide (n = 8) and AP2 inhibitor peptide (n = 8). Data shown is presented as the mean A.U.C. (in arbitrary units; Arb.u.) ± S.E.M. Statistical significance was determined using one-way ANOVA with Holms-Sidak correction $p < 0.05$; *, $p < 0.01$; **, $p < 0.005$; ***, $p < 0.001$; **** Contralateral Scrambled peptide vs Ipsilateral Scrambled peptide p-value = 0.0020. Contralateral AP2 inhibitor peptide vs Ipsilateral Scrambled peptide p = 0.0001. Contralateral AP2 inhibitor peptide vs Ipsilateral AP2 inhibitor peptide p = 0.0053. Ipsilateral Scrambled peptide vs Ipsilateral AP2 inhibitor peptide p-value = 0.0053. (E) Recovery curves for male mice taken from (C) and fit to an exponential decay equation. Males displayed a robust decrease in tau. (F) von Frey withdraw threshold of contralateral hind paw in a chronic inflammatory pain model in male animals. Data for the scrambled peptide (n = 7) and AP2 inhibitor peptide (n = 7) groups is represented as mean percentage of baseline ± S.E.M. Significance determined using repeated measures 2-way ANOVA with Bonferroni correction. (G) von Frey withdraw threshold of ipsilateral hind paw in a chronic inflammatory pain model in male animals. Data for the scrambled peptide (n = 7) and AP2 inhibitor peptide (n = 7) groups is represented as mean percentage of baseline ± S.E.M. Significance determined using repeated measures 2-way ANOVA with Bonferroni correction $p < 0.05$: * (H – L) Complied behavioral data for female animals during CFA model of chronic inflammatory pain. (H) Thermal sensitivity of female mice injected with either scrambled (n = 8) or AP2 inhibitor (n = 8) peptide. Females injected with AP2 inhibitor peptide showed accelerated recovery compared to scrambled peptide but exhibited a delay compared to male mice. Data is presented as mean PWL ± S.E.M. Significance determined using repeated measures 2-way ANOVA with Bonferroni correction $p < 0.01$; **, $p < 0.005$; ***. Ipsilateral Scrambled peptide vs Ipsilateral AP2 peptide: Day 3 p-value = 0.0143, Day 5 p-value = 0.0306. (I) Recovery A.U.C. for female mice under experimental conditions; scrambled peptide (n = 8) and AP2 inhibitor peptide (n = 8). Data shown is presented as the mean A.U.C. (in arbitrary units; Arb.u.) ± S.E.M. Statistical significance was determined using one-way ANOVA with Holms-Sidak correction $p < 0.05$; *, $p < 0.01$; **, $p < 0.005$; ***, $p < 0.001$; ****. Contralateral Scrambled peptide vs Ipsilateral Scrambled peptide p-value = 0.0015. Contralateral Scrambled peptide vs Ipsilateral AP2 Inhibitor peptide p-value = 0.0015. Contralateral AP2 inhibitor peptide vs Ipsilateral Scrambled peptide p = 0.0008. Contralateral AP2 inhibitor peptide vs Ipsilateral AP2 inhibitor peptide p = 0.0021. Ipsilateral Scrambled peptide vs Ipsilateral AP2 inhibitor peptide p-value = 0.0171 (J) Recovery curves for female mice taken from (H; Days 1 - 13) and fit to an exponential decay equation. Females did not experience a large change in the rate of their recovery following inhibition of endocytosis. (K) von Frey withdraw threshold of contralateral hind paw in a chronic inflammatory pain model in female animals. Data for the scrambled peptide (n = 4) and AP2 inhibitor peptide (n = 4) groups is represented as mean percentage of baseline ± S.E.M. Significance determined using repeated measures 2-way ANOVA with Bonferroni correction. (L) von Frey withdraw threshold of ipsilateral hind paw in a chronic inflammatory pain model in male animals. Data for the scrambled peptide (n = 4) and AP2 inhibitor peptide (n = 4) groups is represented as mean percentage of baseline ± S.E.M. Significance determined using repeated measures 2-way ANOVA with Bonferroni correction. (M) Graph depicting the thermal sensitivity of the ipsilateral paw in male (n = 8; green) and female (n = 8; purple) animals that received the scrambled peptide. Gray line represents mean scrambled peptide ipsilateral paw withdraw threshold from (Fig. 4B). Boxed area corresponds with observed time point of interest in Figure 4E. Data is represented as the mean paw withdrawal ± S.E.M. Significance was determined using a 3-way ANOVA with Fishers Least Significant Difference Post-hoc test.

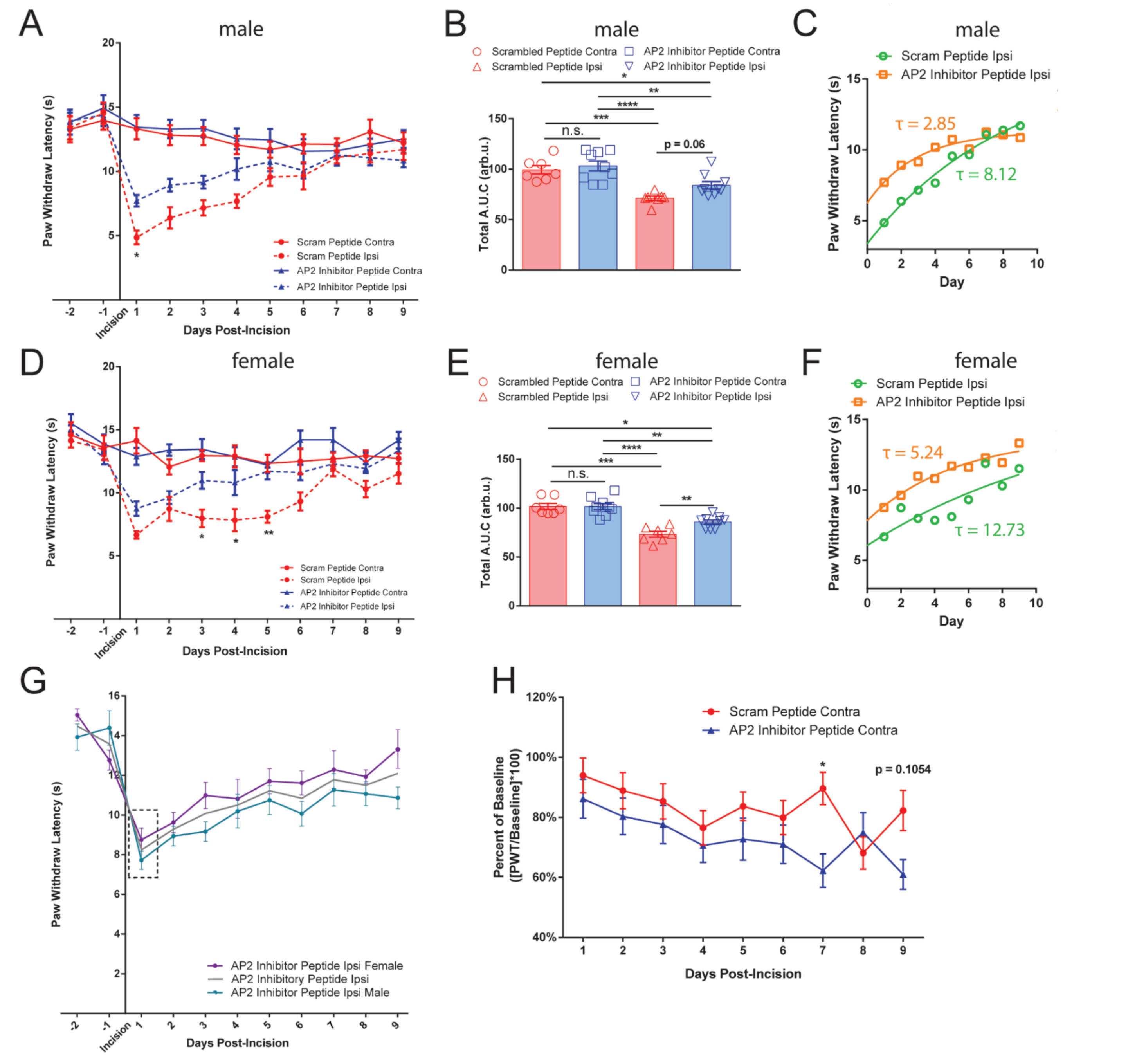

Supplemental Figure 5. Pharmacological inhibition of endocytosis precipitated sexually dimorphic responses to thermal stimuli in a model of post-incisional pain. (A – C) Summarized behavioral data for male rats that underwent the incisional model of post-operative pain. (A) Thermal sensitivity of male rats injected with scrambled (n = 7) and AP2 inhibitor peptide (n = 9) following plantar incision. Males exhibited an early-phase response to local inhibition of endocytosis that was characterized by statistically significant increases in PWT on day 1. Data is presented as mean PWL ± S.E.M. Significance determined using repeated measures 2-way ANOVA with Bonferroni correction $p < 0.05$; *. Ipsilateral Scrambled peptide vs Ipsilateral AP2 peptide Day 1 p-value = 0.0464. (B) A.U.C. quantification for male rats under experimental conditions displayed in (B); scrambled peptide (n = 7) and AP2 inhibitor peptide (n = 9). Data is represented as the mean A.U.C. (in arbitrary units; Arb.u.) ± S.E.M. Statistical significance was determined using one-way ANOVA with Holms-Sidak correction $p < 0.05$; *, $p < 0.01$; **, $p < 0.005$; ***, $p < 0.001$; ****. Contralateral Scrambled peptide vs Ipsilateral Scrambled peptide p-value = 0.0003. Contralateral Scrambled peptide vs Ipsilateral AP2 Inhibitor peptide p-value = 0.0337. Contralateral AP2 inhibitor peptide vs Ipsilateral Scrambled peptide $p < 0.0001$. Ipsilateral Scrambled peptide vs Ipsilateral AP2 inhibitor peptide p-value = 0.0048. (C) Recovery curve (Days 1 – 9) fit to an exponential decay equation. Fitting the recovery curves from (A) reveals that inhibition of endocytosis accelerated the rate of recovery as indicated by a decrease in tau. Male animals responded well to inhibition of endocytosis as indicated by a notable decrease in tau. (D – F) Summarized behavioral data for female rats that underwent the incisional model of post-operative pain. (D) Thermal

sensitivity of female animals injected with scrambled (n = 7) and AP2 inhibitor peptide (n = 9) following plantar incision. Females exhibited a late-phase response to local inhibition of endocytosis that was characterized by statistically significant increases in PWT day 3 – day 5. Data is presented as mean PWL ± S.E.M. Significance determined using repeated measures 2-way ANOVA with Bonferroni correction $p < 0.05$; *, $p < 0.005$; ***. Ipsilateral Scrambled peptide vs Ipsilateral AP2 peptide Day 3 p-value = 0.0125, Day 4 p-value = 0.0137, Day 5 p-value = 0.0016. (E) A.U.C. quantification for female rats under experimental conditions displayed in (B); scrambled peptide (n = 7) and AP2 inhibitor peptide (n = 9). Data is represented as the mean A.U.C. (in arbitrary units; Arb.u.) ± S.E.M. Statistical significance was determined using one-way ANOVA with Holms-Sidak correction $p < 0.05$; *, $p < 0.01$; **, $p < 0.005$; ***, $p < 0.001$; ****. Contralateral Scrambled peptide vs Ipsilateral Scrambled peptide p-value < 0.0001. Contralateral Scrambled peptide vs Ipsilateral AP2 Inhibitor peptide p-value = 0.0015. Contralateral AP2 inhibitor peptide vs Ipsilateral Scrambled peptide $p < 0.0001$. Ipsilateral Scrambled peptide vs Ipsilateral AP2 inhibitor peptide p-value = 0.0081. Contralateral AP2 inhibitor peptide vs Ipsilateral AP2 inhibitor peptide p-value = 0.0011. (F) Recovery curves for females taken from (D) and fit to an exponential decay equation. Female rats injected with the AP2 peptide also exhibited a remarkable decrease in tau compared to their scrambled counterparts. (G) Graph depicting the thermal sensitivity of the ipsilateral paw in male (n = 9; blue) and female (n = 9; purple) animals that received the AP2 inhibitor peptide. Gray line represents mean AP2 inhibitor peptide ipsilateral paw withdraw threshold from (Fig. 5B). Insert box denotes corresponding time point where significance was gained via 3-way ANOVA in **Figure 5**. (H) Dynamic von Frey filament testing of rats in an incisional model of post-operative pain. Data is represented as mean PWT (as a percentage of baseline PWT) ± S.E.M. Statistical significance was determined using repeated measures 2-way ANOVA statistical test with Bonferroni correction $p < 0.05$. Data is complimentary to data presented in figure 5H.

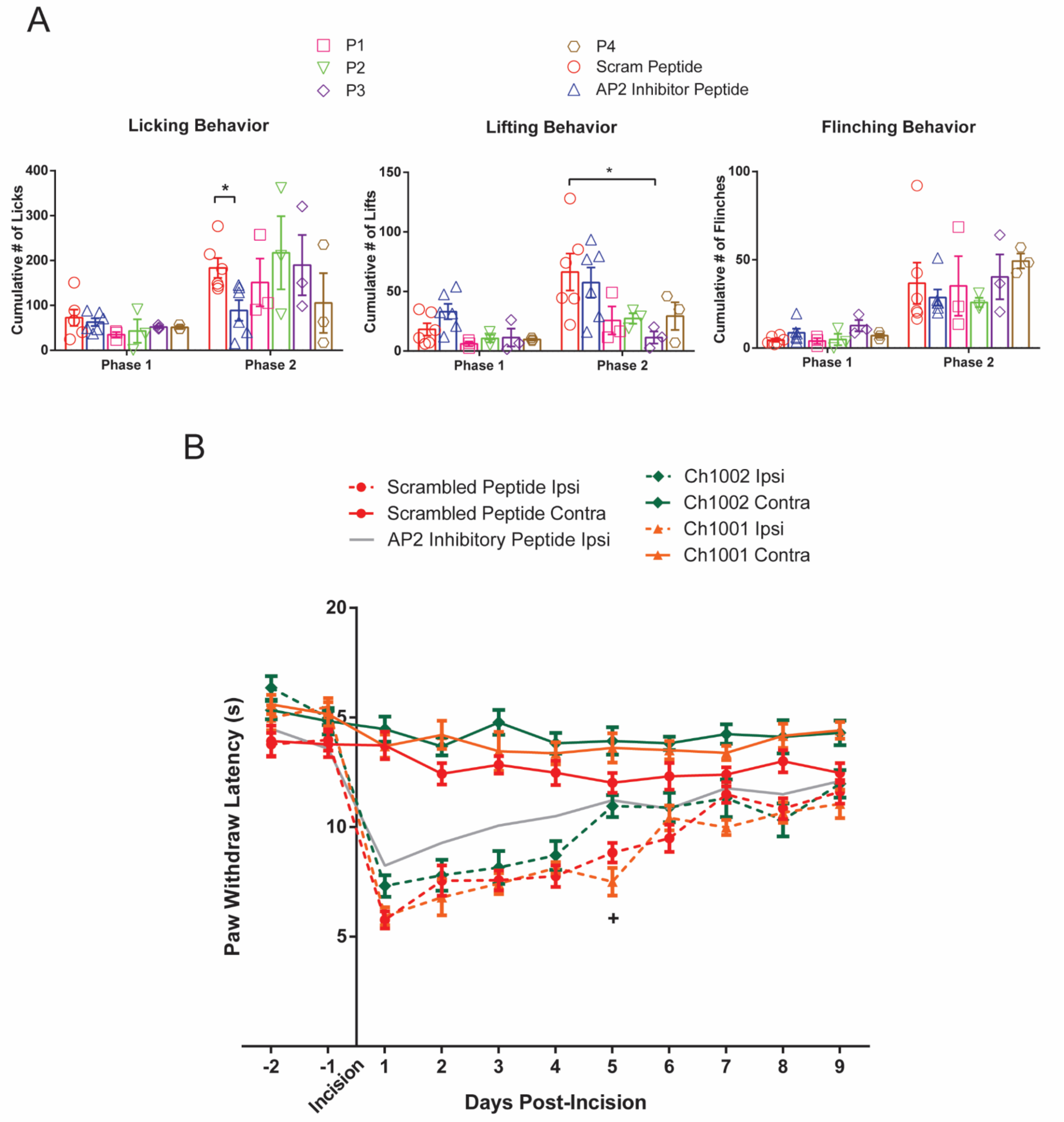

Supplemental Figure 6. Efficacy of analgesia is dependent upon peptide sequence. (A) Summarized pain-like behaviors from C57BL/6 mice following injection with 5% formalin. Phase 1; 0-10 minutes, phase 2; 11-60 minutes post-injection. Scrambled peptide group (n = 6) and AP2 inhibitor peptide group (n = 6) are, respectively, superimposed from Fig. 4A. P1 group n = 3; P2 group n = 3; P3 group n = 3; P4 group n = 3. Peptide sequences found in Supplemental Table 1. Data is presented as cumulative means ± S.E.M. Significance determined using multiple t-tests with Holm-Sidak correction $p < 0.05$; * Phase 2 Licking behavior Scram Peptide vs AP2 Peptide p-value = 0.1402. Phase 2 Lifting behavior Scram Peptide vs P3 p-value = 0.0471. (B) Summarized thermal sensitivity of male rats injected with different $Na_V1.8$ targeted peptidomimetics denoted: Ch1001 (n = 10) and Ch1002 (n = 10) (from Pryce et al., ref 17). Light gray line denotes AP2 peptidomimetic mean as a reference. All data is represented as mean ± S.E.M. and analyzed with 2-way ANOVA with

Bonferroni correction, $p < 0.05$; +. '+' Denotes statistical significance between Ch1002 ipsilateral and scrambled ipsilateral groups; Day 5 p-value = 0.0130.

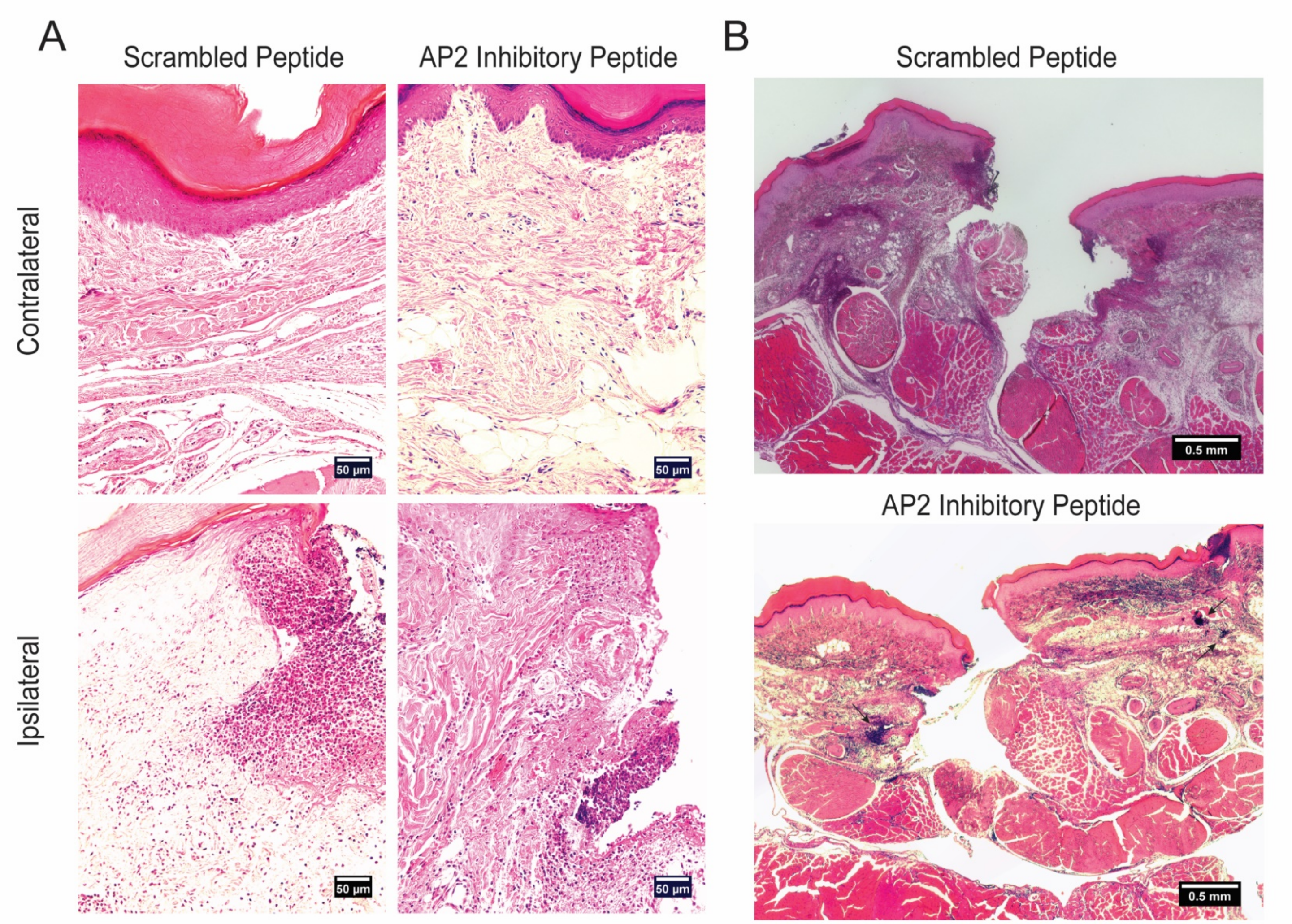

Supplemental Figure 7. Local inhibition of endocytosis did not alter immune cell recruitment but did produce granuloma-like artifacts in an incisional pain model. (A) Animals were treated as previously stated in the Methods section. However, no behavior was collected, instead, animals were sacrificed via transcardial perfusion, and tissue was collected for staining. (*Top*) Representative images depicting hematoxylin & eosin staining of rat hind paws in scrambled (n = 2) and AP2 inhibitor peptide groups (n = 2). Localized immune cells can be observed in the dermis in each condition. (*Bottom*) 24 hours following incision, there is a rapid increase in the number of immune cells. (*Bottom right*) Inhibition of endocytosis resulted in dense clustering of immune cells. Injection of the peptide did not potentiate any observable changes in gait. (B) Representative images of incision site in animals that received either the scrambled peptide (*top*) or AP2 inhibitor peptide (*bottom*). *Black arrows* highlight significant granuloma-like structures.

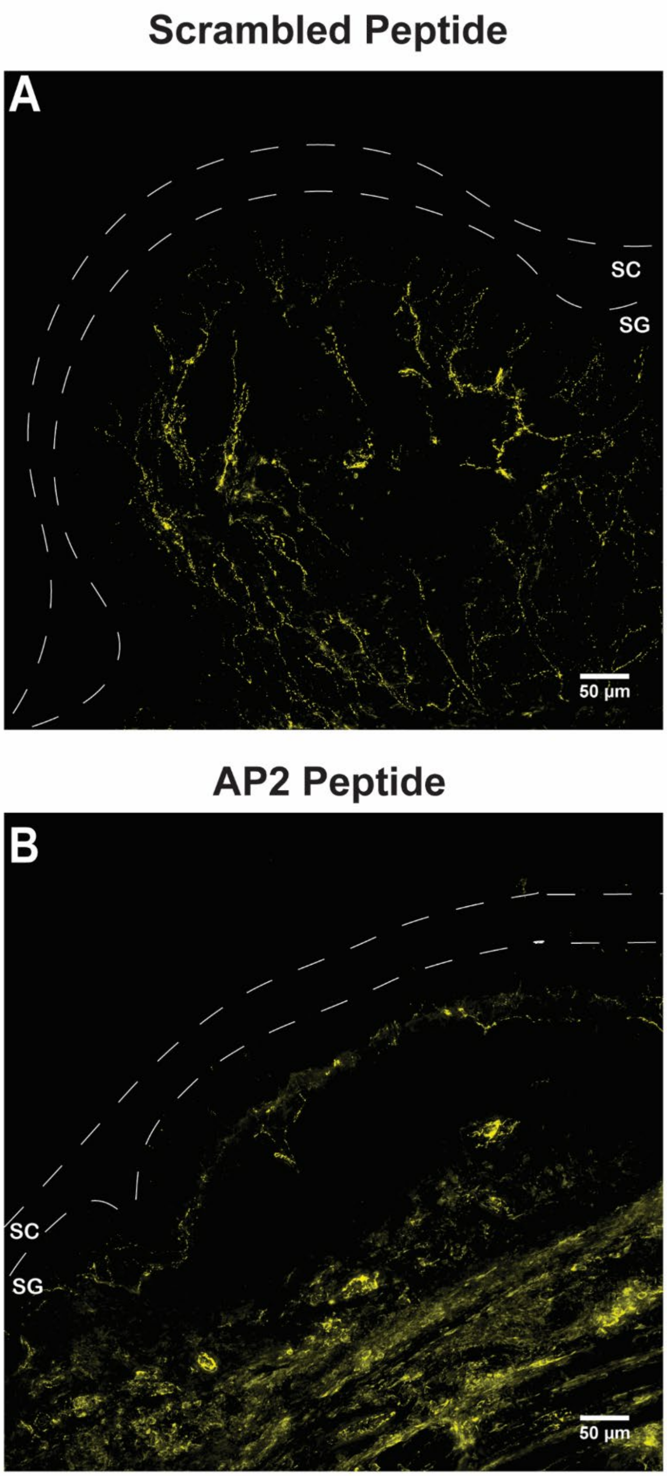

Supplemental Figure 8. Inhibition of endocytosis did not significantly change dermal CGRP immunoreactivity in inflamed paws. (A) Representative image showing CGRP immunoreactivity in a 24 hour CFA-induced inflamed hind paw injected with the scrambled peptidomimetic. (B) Representative image showing CGRP immunoreactivity in an inflamed hind paw injected with the AP2 inhibiting peptidomimetic. SC = Stratum Corneum. SG = Stratum Granulosum
