# Supplementary material for: Inhibiting endocytosis in CGRP+ nociceptors attenuates inflammatory pain-like behavior
Source: Nat Commun. 2021 Oct 4;12:5812. doi: 10.1038/s41467-021-26100-6 (PMC8490418; doi:10.1038/s41467-021-26100-6)
Supplement: Supplementary file 3 — Description of Additional Supplementary Files [file 41467_2021_26100_MOESM3_ESM.pdf]

Description of Additional Supplementary Files

File Name: Supplementary Movie 1
Description: Representative movie depicting the difference in spontaneous pain-like behaviors in animals that received either the Scrambled or AP2α2-targeted shRNA.

File Name: Supplementary Movie 2
Description: Representative movie depicting the difference in spontaneous pain-like behaviors in animals that received either the Scrambled or AP2-inhibitor lapidated peptide.
