# Supplementary material for: Inhibiting endocytosis in CGRP+ nociceptors attenuates inflammatory pain-like behavior
Source: Nat Commun. 2021 Oct 4;12:5812. doi: 10.1038/s41467-021-26100-6 (PMC8490418; doi:10.1038/s41467-021-26100-6)
Supplement: Supplementary file 6 — Reporting Summary [file 41467_2021_26100_MOESM6_ESM.pdf]

nature research

Corresponding author(s): Arin Bhattacharjee

Last updated by author(s): Aug 15, 2021

Reporting Summary

Nature Research wishes to improve the reproducibility of the work that we publish. This form provides structure for consistency and transparency in reporting. For further information on Nature Research policies, see our Editorial Policies and the Editorial Policy Checklist.

Statistics

For all statistical analyses, confirm that the following items are present in the figure legend, table legend, main text, or Methods section.

n/a	Confirmed	
☐	☒	The exact sample size (*n*) for each experimental group/condition, given as a discrete number and unit of measurement
☐	☒	A statement on whether measurements were taken from distinct samples or whether the same sample was measured repeatedly
☐	☒	The statistical test(s) used AND whether they are one- or two-sided<br>*Only common tests should be described solely by name; describe more complex techniques in the Methods section.*
☐	☒	A description of all covariates tested
☐	☒	A description of any assumptions or corrections, such as tests of normality and adjustment for multiple comparisons
☐	☒	A full description of the statistical parameters including central tendency (e.g. means) or other basic estimates (e.g. regression coefficient) AND variation (e.g. standard deviation) or associated estimates of uncertainty (e.g. confidence intervals)
☐	☒	For null hypothesis testing, the test statistic (e.g. *F*, *t*, *r*) with confidence intervals, effect sizes, degrees of freedom and *P* value noted<br>*Give P values as exact values whenever suitable.*
☒	☐	For Bayesian analysis, information on the choice of priors and Markov chain Monte Carlo settings
☒	☐	For hierarchical and complex designs, identification of the appropriate level for tests and full reporting of outcomes
☒	☐	Estimates of effect sizes (e.g. Cohen's *d*, Pearson's *r*), indicating how they were calculated

Our web collection on statistics for biologists contains articles on many of the points above.

Software and code

Policy information about availability of computer code

Data collection	Recording of animals during formalin experimentation was conducted using Active WebCam software, version 11.6, published by PY Software. Immunofluorescent images were collected using LAS X software, version 3.7.3.23245, published by Leica Microsystems. Dynamic weight bearing data was collected using the BIOSEB DWB-2 software, version 2.0.63, published by BIOSEB.
Data analysis	Statistical analysis and graph generation was conducted using Prism 6 and Prism 9 (for 3-way ANOVA calculation) published by GraphPad. Immunofluorescent and chemiluminescent image analysis was conducted using ImageJ software, version 1.53c, published by the NIH. Spontaneous pain-like behavior data was compiled in Excel 365, apart of the Microsoft Office 365 suite version 18.2106.12410.0, published by Microsoft.

For manuscripts utilizing custom algorithms or software that are central to the research but not yet described in published literature, software must be made available to editors and reviewers. We strongly encourage code deposition in a community repository (e.g. GitHub). See the Nature Research guidelines for submitting code & software for further information.

Data

Policy information about availability of data

All manuscripts must include a data availability statement. This statement should provide the following information, where applicable:

- Accession codes, unique identifiers, or web links for publicly available datasets
- A list of figures that have associated raw data
- A description of any restrictions on data availability

The data that supports the findings published in this study are available in the source data file readily available with the manuscript.

Field-specific reporting

Please select the one below that is the best fit for your research. If you are not sure, read the appropriate sections before making your selection.

☒ Life sciences ☐ Behavioural & social sciences ☐ Ecological, evolutionary & environmental sciences

For a reference copy of the document with all sections, see nature.com/documents/nr-reporting-summary-flat.pdf

Life sciences study design

All studies must disclose on these points even when the disclosure is negative.

Sample size	For behavioral experiments, sample size was determined via power analysis. Given preliminary data, a power analysis indicated that a sample size of 8 was sufficient to observe a statistical difference between the experimental groups with an alpha = 0.1 and power = 0.9. Sample sizes for electrophysiological experiments were not predetermined, but were determined by the number of viable cells in each well that were healthy enough for recording. In electrophysiological experiments, each cell functions as an independent "n". By recording from as many healthy cells as possible from multiple dishes, this generates data that is sufficient for statistical analysis. Sample sizes for immunofluorescent and chemiluminescent images were set at 3 as a rigorous benchmark that allowed to verify if an observed trend was a random event, or an observed reproducible phenomena, while also minimizing the number of animals used for these experiments. At a sample size of 3, we were able to conduct experimental replicates and observe the impacts of our manipulations in samples taken form 3 individual animals. This encompasses all experiments presented in the body of the manuscript.
Data exclusions	No data was excluded in this study.
Replication	Each experiment containing chemiluminescent or immunofluorescent images were conducted in triplicate to generate technical replicates; experiments run 3 times using the same samples in randomized order to minimize technical errors, additionally, each experiment was conducted, independently, 3 times to generate experimental replicates; these experiments were conducted using new subjects each time and are used for data analysis. All replications were given there were no technical errors. In the event of a technical error, or the degradation of a reagent, fresh reagents were acquired and the experiment was re-run to see if there were any changes in the previously observed effect.
Randomization	Samples were randomized during image acquisition (immunofluorescence) and sample loading (western blot). Animals were randomized upon arrival and were randomly assigned to treatment groups immediately before receiving treatment.
Blinding	For the formalin assays, scorers were blinded to the phenotype of the animals they were examining. In all other experiments, the experimenter partially blinded to the treatments, but was unblinded to the phenotypes of the animals at the conclusion of the experiment for proper sample collection and sample analysis.

Reporting for specific materials, systems and methods

We require information from authors about some types of materials, experimental systems and methods used in many studies. Here, indicate whether each material, system or method listed is relevant to your study. If you are not sure if a list item applies to your research, read the appropriate section before selecting a response.

Materials & experimental systems

n/a	Involved in the study	
☐	☒	Antibodies
☐	☒	Eukaryotic cell lines
☒	☐	Palaeontology and archaeology
☐	☒	Animals and other organisms
☒	☐	Human research participants
☒	☐	Clinical data
☒	☐	Dual use research of concern

Methods

n/a	Involved in the study	
☒	☐	ChIP-seq
☒	☐	Flow cytometry
☒	☐	MRI-based neuroimaging

Antibodies

Antibodies used	AP2α2: Abcam, ab220065, Rabbit Polyclonal, Lots – GR3218991-2, GR3218991-4, GR3218991-7<br>CGRP: Abcam, ab81887, Mouse Monoclonal, Lot – GR3283855-2<br>HA-Tag: Abcam, ab130275, Mouse Monoclonal, Lot – GR3243470-15<br>Actin: Sigma, A2066, Rabbit Polyclonal, Lot – 019M4777V<br>Goat anti-Rabbit HRP: Promega, W401B, Goat Polyclonal Lot – 0000212738, 0000355714<br>Goat anti-Rabbit 546: Invitrogen, A11035, Goat Polyclonal, Lot – 53423A<br>Donkey Anti-Mouse 488: Abcam, ab150105, Donkey Polyclonal<br>Goat anti-Mouse 555: Abcam, ab150114, Goat Polyclonal, Lot –GR3246256-2
Validation	The primary AP2α2 antibody was validated via unilateral shRNA-mediated knockdown in dorsal root ganglion neurons, Confirmation

of knockdown, and thus antibody validation, was conducted using immunofluorescent and chemiluminescent techniques (figure 1). This antibody has also been previously cited in the following publication: Chen C et al. The Listeriolysin O PEST-like Sequence Co-opts AP-2-Mediated Endocytosis to Prevent Plasma Membrane Damage during Listeria Infection. Cell Host Microbe 23:786-795.e5 (2018). Other primary antibodies have extensive use in various applications as determined by the number of references that specifically cite each antibody; CGRP 60 references and HA-tag 36 references. It is important to note that the HA-tag antibody used in this study has been discontinued due to concerns surrounding the ethical nature of the ascites method for antibody extraction. The rabbit anti-actin antibody has wide use in the literature. The following is a short, but not all inclusive, list of publications that have used this antibody: The zebrafish runzel muscular dystrophy is linked to the titin gene.

Leta S Steffen et al.

Developmental biology, 309(2), 180-192 (2007-08-07), Mutation-specific effects on thin filament length in thin filament myopathy.

Winter JM, et al., The spectraplakin short stop is an actin--microtubule cross-linker that contributes to organization of the microtubule network. Applewhite D A, et al.

Molecular Biology of the Cell, 21(10) (2010), and Pharmacological interrogation of TrkA-mediated mechanisms in hippocampal-dependent memory consolidation. Sylvia Josephy-Hernandez et al. PloS one, 14(6), e0218036-e0218036 (2019-06-25)

Annals of Neurology, 79(6), 959-969 (2016)

Eukaryotic cell lines

Policy information about cell lines

Cell line source(s)	Chinese Hamster Ovarian Cells were obtained from ATCC. Primary dorsal root ganglion neurons were extracted from embryonic-day 15 rat embryos, whereas dissociated dorsal root ganglion neurons were obtained from adult mice.
Authentication	Dorsal root ganglion neuron cultures were validated using electrophysiology. CHO cell lines were not validated.
Mycoplasma contamination	Cell lines were not tested for mycoplasma contamination.
Commonly misidentified lines (See ICLAC register)	No commonly misidentified cell line was used in this study.

Animals and other organisms

Policy information about studies involving animals; ARRIVE guidelines recommended for reporting animal research

Laboratory animals	All mice used in this study are C57BL/6 strain and were purchased from Envigo. Male and female mice used in this study were 8 weeks old. For experiments that required rats, male and female Sprague-Dawley rats were purchased form Envigo. These animals were not age matched, but were matched by weight instead. Pregnant Sprague-Dawley rats were purchased from Envigo on gestational embryonic-day 13. All animal housing rooms held an ambient temperature of 72 degrees farenheit and the humidity was kept at 54%.
Wild animals	This study did not involve wild animals.
Field-collected samples	No field-collected samples were used in this study.
Ethics oversight	All animal experimentation was conducted in accordance with the guidelines set by the “Guide for the Care and Use of Laboratory Animals” provided by the National Institute of Health. All animal protocols were reviewed and approved by the UB Institute Animal Care Use Committee.

Note that full information on the approval of the study protocol must also be provided in the manuscript.
